# An analytical method reduces noise bias in motor adaptation analysis

**DOI:** 10.1038/s41598-021-88688-5

**Published:** 2021-04-29

**Authors:** Daniel H. Blustein, Ahmed W. Shehata, Erin S. Kuylenstierna, Kevin B. Englehart, Jonathon W. Sensinger

**Affiliations:** 1 Department of Psychology and Neuroscience Program, Rhodes College, Memphis, TN USA; 2 Department of Medicine, Faculty of Medicine and Dentistry, University of Alberta, Edmonton, AB Canada; 3 Institute of Biomedical Engineering and Department of Electrical and Computer Engineering, University of New Brunswick, Fredericton, NB Canada

**Keywords:** Computational neuroscience, Motor control

## Abstract

When a person makes a movement, a motor error is typically observed that then drives motor planning corrections on subsequent movements. This error correction, quantified as a trial-by-trial adaptation rate, provides insight into how the nervous system is operating, particularly regarding how much confidence a person places in different sources of information such as sensory feedback or motor command reproducibility. Traditional analysis has required carefully controlled laboratory conditions such as the application of perturbations or error clamping, limiting the usefulness of motor analysis in clinical and everyday environments. Here we focus on error adaptation during unperturbed and naturalistic movements. With increasing motor noise, we show that the conventional estimation of trial-by-trial adaptation increases, a counterintuitive finding that is the consequence of systematic bias in the estimate due to noise masking the learner’s intention. We present an analytic solution relying on stochastic signal processing to reduce this effect of noise, producing an estimate of motor adaptation with reduced bias. The result is an improved estimate of trial-by-trial adaptation in a human learner compared to conventional methods. We demonstrate the effectiveness of the new method in analyzing simulated and empirical movement data under different noise conditions.

## Introduction

Our daily movements, such as reaching for a coffee mug, shooting a basketball, or typing on a keyboard, are constantly being adjusted based on perceived motor performance. If one misses a basketball shot wide right, the next shot will, on average, end up farther left (hopefully closer to the basket). Adaptation of the motor output of the nervous system is shaped by perceived errors. Measuring the adaptation relationship between changes in motor output and perceived errors provides insight into how humans learn to move and how they rely on different information sources in crafting those movements^1,2^.

This adaptation relationship can be calculated by measuring changes in motor output in response to perturbations. However, the corresponding experiments require people to complete movements under abnormal conditions, such as moving through force fields^3–5^, moving with shifted visual feedback^6,7^, or moving with occasional and unpredictable externally imposed disruptions^8,9^. This work has provided significant insight into motor adaptation processes but presents limitations. The experimental manipulations are difficult to implement outside of the laboratory and it remains unclear whether the observed phenomena are relevant to everyday movements. Further, some of these studies require extremely high numbers of repetitive movements.

Trial-by-trial adaptation analysis is an alternative approach to measure this relationship that focuses on error adaptation in unmanipulated environments. This method observes the magnitude of error correction during sequential movements^10–14^, and provides similar insight^10^ as autocorrelation analysis^15^. Trial-by-trial analysis can be run on any type of goal-directed movement under real-world conditions, not relying on applied perturbations. The resulting trial-by-trial adaptation rate has been thought to provide an intuitive metric capturing important motor system characteristics, specifically the relative trust level in control signal generation versus sensory observation^16^. Furthermore, empirical observations in this paradigm have been extensively described with computational models including state-space models^17,18^ and Bayesian models^1,11,14^.

**Figure 1:**
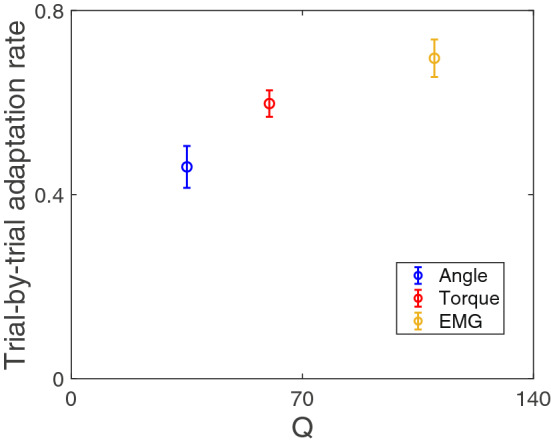
Conventional trial-by-trial adaptation rates increase with increased control noise. Publicly available data^25^ from a movement task with three different controllers of different noise levels. Regression-based trial-by-trial adaptation rates increase with controllers that have increased control noise. Figure adapted from^11^ to display absolute values of trial-by-trial adaptation rates and omit amputee participant data. In the originating study, the control noise parameter of a hierarchical Kalman filter model was tuned to fit experimental data to generate the estimates of control noise for each controller type presented on the x-axis^11^.

Calculation of trial-by-trial adaptation, however, is easily corrupted by unmeasurable noise within the control signal^19,20^, leading to estimates of adaptation that are qualitatively counterintuitive and quantitatively biased^21^. For example, if a person knows their motor output has more noise, they should adapt less to an error. But estimations of trial-by-trial adaptation produce calculations suggesting that they adapt more^11^ (Fig. 1). To date, approaches to estimate trial-by-trial adaptation rates have used an autocorrelation analysis^22,23^ or a linear regression analysis^10,11,14^ that ignored, by averaging, the effect of stochastic variables at play in a motor system, namely the motor control noise and sensory feedback noise^24^. These techniques are sensitive to the noise in the system, such that they produce counterintuitive and biased estimations. One attempt successfully reduced overall estimation bias using an Adjusted Yule-Walker method on simulated data, but the approach requires large numbers of trials to be analyzed and produces highly variable estimates^21^. The theoretical promise of calculating unbiased trial-by-trial adaptation remains, and in this work we propose a novel and broadly applicable method to reduce the inherent bias in estimates of the trial-by-trial adaptation rate.

**Table 1:** Summary of variable definitions.

Variable Name	Definition—Descriptive	Definition—Expression
${\eta }_{q}$	Control noise with variance $Q$	${\eta }_{q}=N(0,Q)$
${\eta }_{r}$	Sensory feedback noise with variance $R$	${\eta }_{r}=N(0,R)$
${\eta }_{\xi }$	Internal model noise	${\eta }_{\xi }=N(0,\xi )$
$B$	System dynamics	actual parameters, e.g. controller gain
$\widehat{B}$	Estimated system dynamics	$\widehat{B}=f(params)$, from estimated parameters
$u$	Motor command	$u= \frac{{x}_{i}}{\widehat{B}}$
${x}_{i}$	Intended target	
${x}_{u}$	Result of $u$ in noise-free environment	${x}_{u}=B\cdot u$
${x}_{m}$	Measurable endpoint of movement	${x}_{m}={x}_{u}{+ \eta }_{q}$
${x}_{s}$	Sensed endpoint of movement	${x}_{s}={x}_{m}{+ \eta }_{r}$
${x}_{p}$	Posterior estimate of movement endpoint	${x}_{p}={f(x}_{i},{x}_{s},\xi )$

Since it is impossible to externally measure motor noise on a trial-by-trial basis, we sought to estimate the true adaptation rate indirectly. In this work we first demonstrate that the conventional calculation of trial-by-trial adaptation rate, even under steady-state conditions, produces paradoxical results likely due to the overlying noise. Then we present a novel approach to estimate an unbiased trial-by-trial adaptation rate using a model-free analytic stochastic method to filter out overlapping noise. We compare the conventional and proposed method using simulated and empirical data collected with a simple movement experiment. The proposed analytic technique provides a closer approximation to the elusive motor parameters of interest, providing a more useful error adaptation measurement that is relevant for unperturbed movements across a variety of contexts. We conclude with suggestions for how to use the new analysis tool and discuss the wide-ranging research and clinical applications in supporting informative motor assessment.

## Results

**Figure 2:**
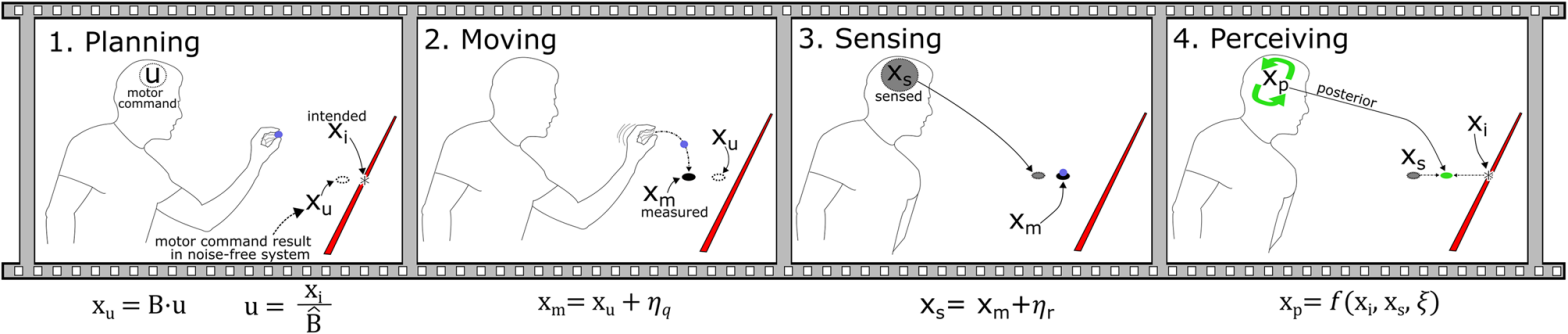
Overview of the movement generation framework during a task of tossing a ball to hit a target. In the Planning phase, the thrower generates a motor command $(u)$ that, in a noise-free environment, will result in a specific ball landing point $\left({x}_{u}\right).$ In other words, a control action $u$ is formed using an inverse model of the user's estimate of system dynamics $(\widehat{f}(u))$, and ${x}_{u}$ is obtained by propagating this action $u$ through the actual dynamics $f$: [${x}_{u}{=f({\widehat{f}}^{-1}(x}_{i}))$]. The difference between ${x}_{u}$ and the intended target $({x}_{i})$ represents misestimation of system parameters that are continually updated through the learning process. In the Movement phase, the throw is completed with ${x}_{u}$ being affected by *control noise* $({\eta }_{q})$ to produce the actual measurable position $({x}_{m})$. In the Sensing phase, the actual movement endpoint $({x}_{m})$ is corrupted by *feedback noise* $({\eta }_{r}$), resulting in the endpoint sensed by the thrower $({x}_{s})$. In the Perceiving phase, a posterior estimate $({x}_{p})$ of the landing point is arrived at by combining information from the intended endpoint $\left({x}_{i}\right)$, the sensed endpoint $({x}_{s})$, and the level of internal model noise $(\xi )$^1,11^.

Trial-by-trial Adaptation Rate (AR) is broadly defined as the ratio between the trial-to-trial change in movement endpoint and trial error:

1 $$AR= \frac{{x}^{k+1}- {x}^{k}}{{x}^{k}- {x}_{i}}$$

where superscript $k$ denotes a given trial number and ${x}_{i}$ denotes the intended target. Note that the target modality may be any continuous variable such as position, force, sound frequency, etc. We use the term position throughout the manuscript, but the concepts apply to any signal modality. The generic position $(x)$ terms do not adequately differentiate between the various positions, which include the intended position $\left({x}_{i}\right)$, the motor command result in a noise-free system $\left({x}_{u}\right)$, the externally measured position $\left({x}_{m}\right)$, the sensed position $\left({x}_{s}\right)$, and the perceived position $\left({x}_{p}\right)$. See Table 1 for an overview of variable definitions and Fig. 2 for a depiction of how these variables are used to mathematically represent the movement generation process.

When the various types of position are delineated, we can better articulate the implicit assumption that adaptation refers to the response of the person’s intent or noise-free actions (i.e. ${x}_{u}$ domain), in response to their perceived error (i.e. ${x}_{p}$ domain). We may accordingly use these domains within our adaptation equation. Although impossible to measure empirically, this definition serves as our *gold* standard, defined as:

2 $${AR}_{Gold}= \frac{{x}_{u}^{k+1}- {x}_{u}^{k}}{{x}_{p}^{k}- {x}_{i}},$$

Pragmatically, neither the ${x}_{u}$ domain nor the ${x}_{p}$ domain can be measured, and so conventional definitions use the ${x}_{m}$ domain as a proxy for both:

3 $${\widehat{AR}}_{Conv}= \frac{{x}_{m}^{k+1}- {x}_{m}^{k}}{{x}_{m}^{k}- {x}_{i}}$$

This rate is typically calculated using a linear regression^10,14,26^ or auto-correlation analysis^22,23^. Note the use of ${x}_{i}$, the intended movement endpoint, which we consider equivalent to ${x}_{T}$, the externally dictated target. In constrained experiments as presented here, these should be the same. These terms may differ when external constraints are present such as movements to a target close to a cliff where an overshoot error would have greater negative consequences than an undershoot error.

**Figure 3:**
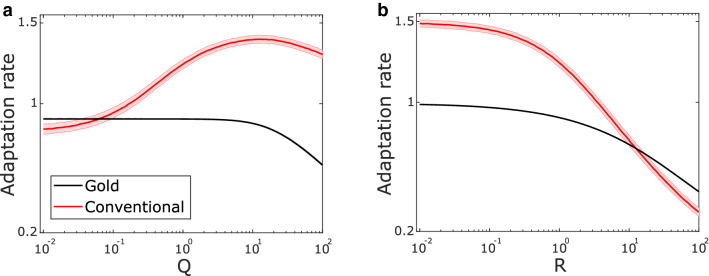
Conventional trial-by-trial adaptation does not capture the expected dynamics of human motor performance. (**a**) Simulated adaptation rate values with changing control noise (Q). The shaded area indicates one standard deviation above and below the mean (solid line). Results from 1,000 simulations at each of 100 values of Q across the range indicated with ${x}_{i}=100$, $R=1$ and $\xi =0.01$. (**b**) Simulated adaptation rate values with changing sensory noise (R). Simulation settings and parameters as in a except here $Q=1.$ All adaptation rates are shown as absolute values.

To compare the gold standard (Eq. 2) with the conventional regression analysis (Eq. 3), we generated simulated movement data across a range of simulation parameters using a Bayesian model of an experienced performer in which trial-by-trial adaptation depends on the subject’s true knowledge of control noise, sensory noise, and internal model confidence (see Methods) (Fig. 3). For increasing control noise (Fig. 3a), the modelled learner should adapt less. The gold-standard definition accurately captures this phenomenon (Fig. 3a, black line). Surprisingly, the conventional estimation shows a qualitatively different phenomenon—its estimate increases as control noise increases (Fig. 3a, red line). For increasing sensory noise (Fig. 3b), the qualitative trend between the two metrics is the same, though the quantitative bias is substantial and qualitatively wrong (an adaptation rate greater than 1 indicates overcompensation).

The gold standard definition and conventional estimation differ in the domains used in both their numerator and denominator, and this counterintuitive trend could be caused by either one or a combination of both. As we analytically explain below, using the proxy for the numerator does result in qualitative and quantitative problems. In contrast, using the proxy for the denominator does not produce counterintuitive effects (increases in ${\eta }_{q}$ or ${\eta }_{r}$ lead to increases in estimated adaptation rate, and increases in $\xi$ lead to decreases in estimated adaptation rate, as we would expect) and only mild biases. It is thus important not to use the proxy in the numerator, but less important to avoid the proxy in the denominator. Pragmatically, there is no way to recover ${x}_{p}$ from ${x}_{m}$ without relying on model-based assumptions, but we can recover ${x}_{u}$ from ${x}_{m}$ using simple stochastic analysis, as we show below.

We accordingly define a silver-standard proxy for the adaptation rate as follows:

4 $$A{R}_{silver}= \frac{{x}_{u}^{k+1}- {x}_{u}^{k}}{{x}_{m}^{k}- {x}_{i}}$$

**Figure 4:**
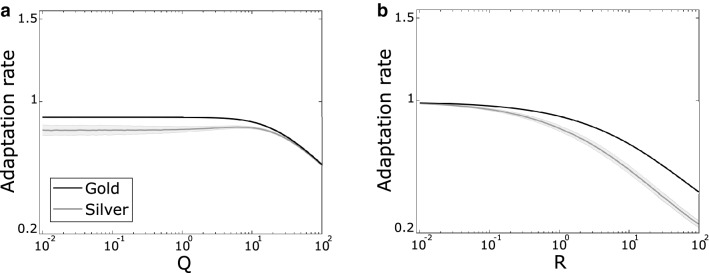
Silver adaptation rates approximate gold standard adaptation rates. Simulated data for changing control noise (**a**) and sensory noise (**b**). Silver adaptation rates can be calculated from data collected in carefully controlled experiments ^4^. Simulation parameters as in Fig. 3.

This *silver* definition closely tracks the gold adaptation rate when applied to simulated data with increasing control noise (Fig. 4a) and sensory noise (Fig. 4b). This definition can be calculated under carefully controlled experimental conditions where the control noise added on each trial is known in order to calculate ${x}_{u}{=x}_{m}-{\eta }_{q}$, which is the case in the empirical motor study included in this work. The silver adaptation rate can accordingly be used as a baseline against which to compare conventional estimation along with the proposed new estimation approach.

**Figure 5:**
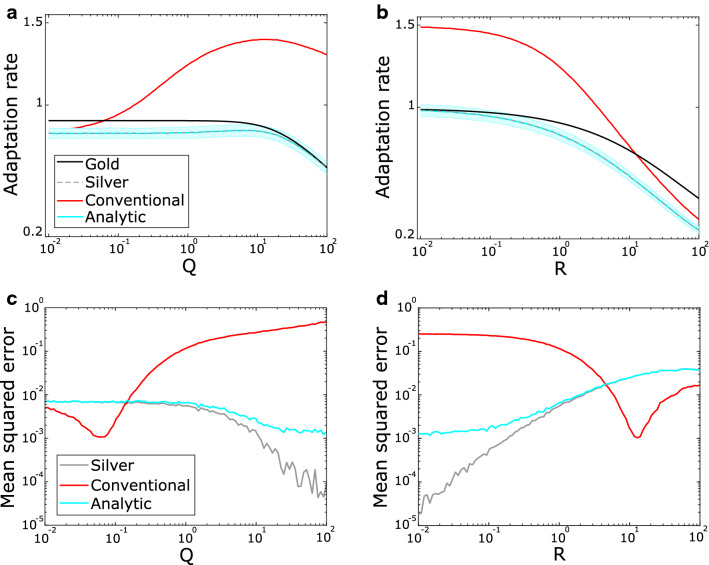
Comparison of different trial-by-trial adaptation rate calculation methods. (**a–b**) Simulation and parameter settings as in Fig. 3 for changing control noise with R = 1 (**a**) and changing sensory noise with Q = 1 (**b**). For clarity, the one standard deviation shaded range is shown only for the analytic results (see Figs. 3 and 4 for variability shading for other analysis methods). (**c**, **d**) Performance of each analysis method shown in (**a**) and (**b**) as measured using the mean squared error compared to the gold adaptation rate for changing control noise (**c**) and sensory noise (**d**).

The silver definition provides an experimentally measurable baseline that is in qualitative and quantitative agreement with the gold standard, but it cannot be measured in real-world conditions without applying known perturbations – the very conditions that make trial-by-trial adaptation so appealing. We accordingly sought to develop a way to estimate the true adaptation rate that could be applied to a variety of laboratory and real-world movement data. The analytic approach, using statistical principles to filter out the noise effects and estimate the silver adaptation rate, can be defined as follows (see Methods for derivation):

5 $${\widehat{AR}}_{analytic} = \frac{cov\left({x}_{m}^{k+1},{x}_{m}^{k}\right)- {var(x}_{m}^{k}) + Q}{{var(x}_{m}^{k})},$$

where the variance of control noise $Q$ can be measured or estimated from the experiment. We systematically varied simulation parameters (as in Fig. 3) and compared the analytic estimate to the gold AR, silver AR and the conventional regression estimate (Fig. 5).

The analytic estimate better captures the qualitative and quantitative trends observed for changing control noise compared to the conventional regression estimate (Fig. 5a). We also observed more quantitatively aligned estimates under conditions of changing sensory noise (Fig. 5b). Estimation error plotted across the changing parameters shows that the analytic estimate consistently produces better estimates of the gold adaptation rate than the conventional regression method (Fig. 5c,d). The one exception is that due to the different trends in adaptation rates with changing parameters, the conventional regression happens to overlap with the gold adaptation rate showing a low estimation error indicated by low mean squared error when Q < R (Fig. 5c,d). When internal model noise ($\xi$) was varied we observed similar quantitative improvements using the new method compared to the conventional regression estimate.

**Figure 6:**
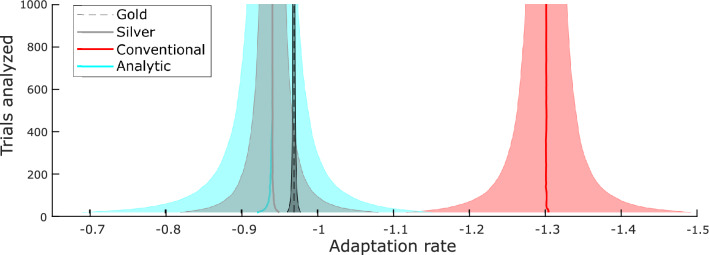
Analyzing more trials reduces variability of results. 10,000 simulations were run with the following parameters: $Q=1$, $R=1$, $\xi =0.1$. Different windows of trials were analyzed on each simulation run with the shaded area indicating one standard deviation of the total results.

Estimation results presented so far involved the analysis of 1,000 trials of simulated movement data, a number not always feasible for everyday experiments. We sought to determine the variability of the estimation methods with different numbers of trials analyzed. We systematically varied the size of the analyzed trial window and observed the performance of each analysis technique on simulated data (Fig. 6). As the number of trials analyzed increases, the variability of each estimation method is reduced (Fig. 6).

**Figure 7:**
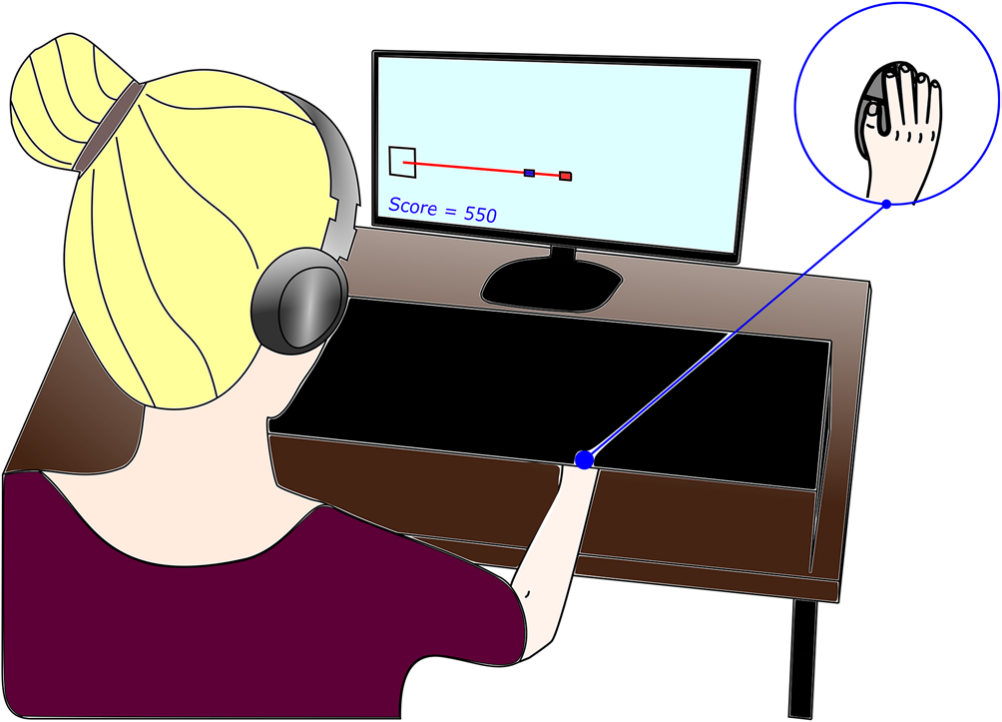
Experimental setup. Participants controlled a blue cursor on a screen using a computer mouse. Endpoint only feedback was provided after each movement was completed. See Methods for detailed description of equipment and protocol.

**Figure 8:**
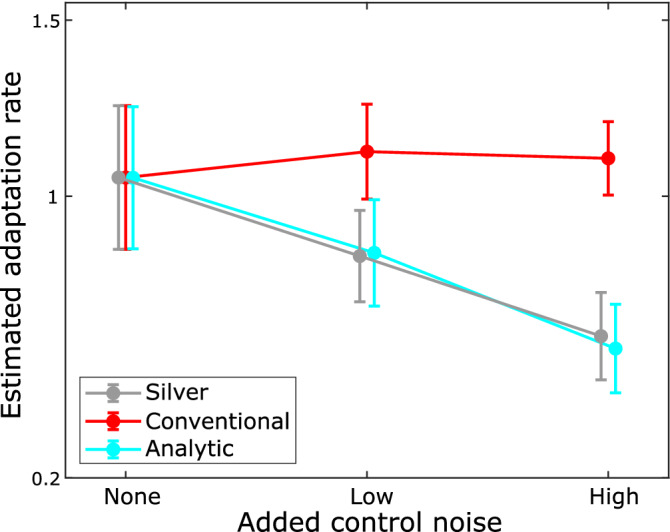
Performance of analysis techniques on empirical data. The means with standard deviation error bars comparing the three analysis methods run on data collected from 26 able-bodied participants completing a computer cursor movement study under different added noise conditions. Note that the silver adaptation rate and conventional estimate are equivalent with no added control noise. All pairwise differences within silver ARs and analytic ARs are statistically significant (ANOVA with Bonferroni-corrected post-hoc comparison, p < .001).

In an empirical study (Fig. 7), we observed a similar improvement in estimated adaptation rates using the analytic method (Fig. 8). Twenty-six able-bodied participants each completed three sets of 100 movements under different noise conditions (the 27^th^ participant was removed as an outlier, see Methods-Analysis for details). Participants moved a mouse cursor on a screen to a visual target and were provided with endpoint landing position feedback only. The order of the three noise conditions – NO added noise, LOW added noise, HIGH added noise – was randomized for each participant. The endpoint error and externally applied noise were recorded for every trial and then used to run the adaptation rate estimation methods.

Increasing levels of control noise resulted in no significant changes in trial-by-trial adaptation rates using the conventional regression approach (Fig. 8, red line). However, as predicted, the silver adaptation rate estimate showed a proportional decrease with increasing control noise (Fig. 8, gray line), a trend closely matched by the analytic estimate (Fig. 8, cyan line).

The data resulting from each analysis method shown in Fig. 8 were analyzed with a separate one-way ANOVA with repeated measures using Bonferroni-corrected post-hoc comparisons:

- For the conventional estimates of trial-by-trial adaptation rate, Mauchly’s Test of Sphericity indicated that the sphericity assumption was not violated $({\upchi }^{2}(2) = 1.976, p=.372$). The repeated measures ANOVA did not indicate statistical significance between estimated adaptation rates across different noise conditions (F(2,50) = 1.676, *p* = 0.197).
- For the silver trial-by-trial adaptation rates, Mauchly’s Test of Sphericity indicated that the sphericity assumption was violated $({\upchi }^{2}(2) = 8.566, p=.014$). The repeated measures ANOVA with Greenhouse–Geisser correction indicated a statistically significant difference of estimated adaptation rates across different noise conditions (F(1.538,38.457) = 65.310, *p* < 0.001). All Bonferroni-corrected pairwise comparisons were statistically significant (*p* < 0.001).
- For the analytic estimate trial-by-trial adaptation rates, Mauchly’s Test of Sphericity indicated that the sphericity assumption was not violated $({\upchi }^{2}(2) = 5.080, p=.079$). The repeated measures ANOVA indicated a statistically significant difference of estimated adaptation rates across different noise conditions (F(2,50) = 76.815, *p* < 0.001). All Bonferroni-corrected pairwise comparisons were statistically significant (*p* < 0.001).

In summary, the conventional adaptation method was not able to capture the clear decrease in adaptation caused by increasing control noise, whereas the analytic method was able to do so, and its estimate closely aligned with the silver definition.

## Discussion

Here we have developed an analytic method to better estimate trial-by-trial adaptation rates. When applied to simulated data, the novel approach produces qualitatively accurate and quantitively improved estimates of the gold adaptation rate, compared to the conventional regression estimate. Following validation with simulated data, we ran an empirical study in which participants moved a cursor on a screen with a computer mouse under varied noise conditions. Analysis of empirical data matched the simulation results: the novel analysis approach produced better adaptation rate estimates compared to the conventional regression estimate. In the empirical work, adaptation estimates were compared to a close approximation of the gold adaptation rate—the silver estimate—a value that can only be calculated under carefully controlled experimental conditions. The analytic adaptation rate estimation method proposed here can be run across a wide range of motor contexts, both within and outside of the laboratory. Due its versatility, accuracy and sensitivity, the new proposed technique provides a path to reduce bias in the analysis of human motor performance.

The estimation method resulting from this work can be applied in the laboratory to advance motor adaptation research in several ways. First, the method provides a well-justified and standardized approach to measure adaptation. In the past there have been different ways to measure adaptation that result in incomparable numerical indicators, e.g. trial-by-trial adaptation and perturbation adaptation^10^. The new method can be applied in a wide range of contexts to allow for direct comparisons across different experimental setups. Second, the method reduces experimental limitations associated with perturbation adaptation studies. These perturbation studies, looking at the magnitude of adaptation to an unexpected externally applied disturbance, avoid some noise biasing associated with trial-by-trial adaptation because the perturbation magnitude dominates the baseline system noise. One issue is that the applied perturbations can alter motor dynamics, interfering with the process being observed. Perturbation adaptation studies also require large numbers of trials because the disturbances can only occur sporadically. The new method eliminates both issues associated with perturbation adaptation studies and other lab-confined approaches such as error clamping.

Our approach resulted in estimates of trial-by-trial adaptation rates with much lower variability compared to the previously described Adjusted Yule-Walker method^21^. Remaining estimation bias was minimized with fewer analyzed trials—after 100 to 200 trials in our work (see Fig. 6) compared to about 400 trials necessary for estimation bias to be minimized using the Adjusted Yule-Walker method^21^. Others have adopted maximum likelihood system identification algorithms to estimate hidden model parameters^27,28^ which can be used to remove the noise bias from adaptation rate estimates. This approach, however, is very sensitive to the model selected, requires large numbers of movement trials, and requires sophisticated laboratory manipulations such as error clamping^28^.

The application of this novel approach to estimate adaptation rate goes beyond the laboratory environment. Since the method operates on unperturbed movement data, it can be run in clinical or real-world settings. For example, a stroke patient could make repetitive reaching movements with an occupational therapist. Understanding the magnitude of error adaptation is critically insightful in such a case, providing details about motor deficiencies and compensatory strategies that are overlooked by currently available clinical motor assessments. Or movement data could be extracted from video recordings of a pitcher in a baseball game; without the need for manipulations or complicated measurements, the adaptation analysis approach can be applied to almost any sequence of movements.

Although the analytic method provides accurate estimates, it does require knowledge of the baseline control noise (Q). This parameter can be measured with a separate experiment in which the learner makes movements in the absence of sensory feedback and the endpoint variability is recorded. Sporadic feedback is necessary to reduce endpoint drift and although removing exteroceptive feedback is straightforward, proprioceptive feedback usually remains. There are other ways to estimate control noise including measurement of the just-noticeable-difference of a perturbation that can be converted to a total noise estimate^29^ with subsequent subtraction of an estimate of sensory noise. Noise can also be estimated without running additional experiments by optimizing particular noise values that result in expected parametric noise properties (such as zero mean), as in Ahn et al.^21^. We plan to explore such methods in future work. Another limitation of the analytic method is that its results show high variability similar to the conventional regression method when low numbers of trials are analyzed (Fig. 6). The improved estimation accuracy is maintained across trial window sizes and we suggest analyzing as many trials as possible.

A few limitations of the validation of the proposed protocol should be addressed. The need for simulated data to test and compare the estimation methods requires the adoption of a specific motor control model that requires assumptions about the underlying computational mechanisms driving motor processes. Here we used a Bayesian model of motor control which has been shown to be supported in many^1,14^ but not all motor contexts^16^. Other models could have been used including state-space only models^17,18^ or cost-function models^30–32^. Nevertheless, the estimation improvements we observed in the empirical data were similar to those observed with simulated data, suggesting the assumptions required to generate simulated motor data have not affected our findings. The primary motivation was in overcoming *qualitative* issues with the conventional regression estimation, further emphasizing that the specific model chosen is not critical. Even if the underlying motor control model to generate simulated data were different, the estimation methods should produce qualitatively similar results. Any effect on the results associated with choosing a different model would be represented by a consistent shift in quantitative results, something we may expect when going from simulated to empirical data anyway.

In collecting empirical data to test the proposed method, a few limitations arose. Since we wanted a precise estimate of control noise and we were comparing against the silver estimate that requires knowledge of the noise added to each movement trial, we focused only on the noise we experimentally added. This approach ignored the baseline control noise inherent in any moving human. Since we were most interested in qualitative trends, we were justified in ignoring baseline noise for the silver and analytic approaches. It is not possible to ignore the baseline noise in the conventional regression approach but since the noise we added was much greater than baseline noise, we were still able to observe and compare qualitative trends.

Another limitation was the possible impact of order effects in our within-subjects experimental design. We randomized the order of conditions for each participant to average out any order effect, with the consequence of potentially noisier results. The averaging out of differences across order conditions could have contributed to the lack of a trend observed in our empirical conventional adaptation rates (red line in Fig. 8) which differed from the trend observed in the external dataset (Fig. 1). However, it is also possible that the adaptation rates in our empirical data span a flatter part of the curve (from Fig. 3), in which case the lack of an increasing trend would not be a surprise.

And finally, differences in sensory noise between participants could have affected the results. We tried to eliminate as much feedback as possible by using noise reducing headphones and by visually occluding the moving arm, but proprioceptive inputs were not eliminated. The ability to interpret proprioceptive inputs to drive motor adaptation will vary for each individual research participant, leading to noisier empirical data that may be differentially impacted across control noise conditions. Again, since the focus was on qualitative trends, these individual differences should not affect the conclusions of the study. In our simulations we were able to keep sensory noise constant and we observed similar results. If sensory noise is a concern to others using these methods, it can be measured using a separate experimental protocol^33^.

Here we have demonstrated that current motor adaptation analysis is biased by noise, and we provide an important methodological advance to correct this issue. The result is a more accurate estimation of trial-by-trial adaptation that better captures what makes this metric useful: the adaptation of *motor intent* based on perceived error. Not only will the analytic adaptation rate estimation solution we provide support improved laboratory analysis and the correction of possible misinterpretation of data in previous studies, but it will allow for the analysis of a wide range of motor behavior in real-world settings. Using trial-by-trial adaptation rates as an informative clinical motor metric is promising, and now possible with the advances presented here.

## Methods

In the following subsections we first present the derivation of the analytic estimation approach and then describe the simulations and experiments we used to assess our techniques.

### Analytic estimation of adaptation rate

The least squares regression of the slope $\beta$ for the linear relationship $y=\alpha + \beta x$ is:

6 $$\widehat{\beta }=\frac{\sum \left({x}_{i}-\overline{x }\right)\left({y}_{i}-\overline{y }\right)}{\sum {\left({x}_{i}-\overline{x }\right)}^{2}}$$

However, the contribution of noise sources in $x$ and $y$ are not apparent from this expression, and as we have shown in the results, it is important to be able to compensate for noise sources in $y$. An equivalent well-known analytic representation may be used, which makes use of stochastic methods to produce a probabilistic estimator:

7 $$\widehat{\beta }= \frac{cov(x,y)}{var(x)}$$

It will be easier to see and remove the contribution of noise sources using this analytic estimation. Applying this analytic estimate of the slope to our silver definition of adaptation rate (Eq. 4), our estimate of AR is:

8 $$\begin{aligned} \widehat{AR}_{analytic} & = \frac{{{\text{cov}} \left( {x_{u}^{k + 1} - x_{u}^{k} ,x_{m}^{k} - x_{i} } \right)}}{{{\text{var}} \left( {x_{m}^{k} - x_{i} } \right)}} \\ & = \frac{{{\text{cov}} ((x_{m}^{k + 1} - \eta_{q}^{k + 1} ) - \left( {x_{m}^{k} - \eta_{q}^{k} } \right), x_{m}^{k} - x_{i} )}}{{{\text{var}} \left( {x_{m}^{k} - x_{i} } \right)}} \\ \end{aligned}$$

Simplifying the numerator,

9 $$\begin{aligned} cov\left( {(x_{m}^{k + 1} - \eta_{q}^{k + 1} ) - \left( {x_{m}^{k} - \eta_{q}^{k} } \right), x_{m}^{k} - x_{i} } \right) & = cov\left( {x_{m}^{k + 1} - q^{k + 1} - x_{m}^{k} + q^{k} , x_{m}^{k} } \right) \\ & = cov\left( {x_{m}^{k + 1} ,x_{m}^{k} } \right) - cov\left( {q^{k + 1} ,x_{m}^{k} } \right) - cov(x_{m}^{k} ,x_{m}^{k} ) + cov\left( {q^{k} , x_{m}^{k} } \right) \\ & = cov\left( {x_{m}^{k + 1} ,x_{m}^{k} } \right) - 0 - var\left( {x_{m}^{k} } \right) + Q \\&= cov\left( {x_{m}^{k + 1} ,x_{m}^{k} } \right) - var\left( {x_{m}^{k} } \right) + Q \\ \end{aligned}$$

Simplifying the denominator,

10 $$var(x_{m}^{k} - x_{i} ) = var(x_{m}^{k} )$$

Combining our simplified numerator and denominator, our estimate accordingly can be calculated as:

11 $$\widehat{AR}_{analytic} = \frac{{cov\left( {x_{m}^{k + 1} ,x_{m}^{k} } \right) - var(x_{m}^{k} ) + Q}}{{var(x_{m}^{k} )}}$$

We call Eq. 11 the *analytic* estimate of the trial-by-trial adaptation rate. It can be calculated from endpoint error records of movements and an estimate of the control noise variance.

### Simulations

#### Simulated data generation

For all simulated data, MATLAB software (MathWorks, Natick, MA, version 2020a) was used to run a hierarchical Kalman filter model that has been described in detail elsewhere^10^. Briefly, the first part of the model uses a Kalman filter to generate a posterior estimate of the endpoint position (i.e. ${x}_{p}$) by fusing the a priori premotor estimate (i.e. ${x}_{i}$) and the post-movement sensory observation (i.e. ${x}_{s}$). The second layer of the model uses another Kalman filter to generate an update in the learner’s estimation of system parameters. In this case we use a single parameter representing the gain of the controller; the learner’s misestimation of the controller gain leads to motor errors. The magnitude of the parameter estimate update is determined by the second Kalman filter’s integration of the perceived error and the overall internal model uncertainty that is driven by the uncertainty increment at each trial $(\xi )$. For example, if the internal model uncertainty is high, parameter estimate updates will be larger.

Simulated data were generated with changing input parameters to test the sensitivity of each estimation method. Each of the three input parameters—$Q$, $R$, and $\xi$—was systematically varied while the other two were held constant. Constant parameters values were as follows: $Q=1$, $R=1$, $\xi =0.1, {x}_{i}=100$. When varied, 100 equally spaced values on a log scale were used for the changing input parameter ranging from 10^–2^ to 10^2^, enabling the parameter to range from being dominated to dominating. For each set of input parameters, 1,000 simulations were run, each with 1,500 movement trials with the last 1000 trials used for analysis. All other parameters were constant including the controller gain ($B=1$), initial estimated gain (${\widehat{B}}^{(1)}=1$), and the initial overall internal model uncertainty (${P}_{p}^{(1)}=5$). The gold, silver, conventional, and analytic adaptation rate estimates were calculated for each simulated dataset. Mean squared errors were computed comparing the estimates to the gold trial-by-trial adaptation rate.

To explore the sensitivity of the estimation methods when run on different numbers of trials, a separate set of simulated data was generated with fixed parameters ($Q=1$, $R=1$, $\xi =0.1$). For each of 10,000 simulation runs, data from 2000 movement trials were generated. Starting at the 1000^th^ trial, the data were analyzed using each estimation procedure across 99 trial windows of varying sizes, equally spaced from 20 to 1000 trials.

### Empirical study

#### Ethics oversight

All research with human participants was conducted with approval and oversight by the Rhodes College Institutional Review Board. All participants provided written informed consent. All methods were conducted in accordance with relevant guidelines and regulations.

#### Participants

27 able-bodied and right-handed (self-reported) participants [mean age = 18.7 yrs, range = 18–21, 23 females], recruited from the Rhodes College Psychology Department participant pool, completed the experiment. Two additional participants did not complete the full experiment and their data were not included. Participants were compensated for their time with class credit points.

#### Setup

While seated comfortably at a desk and viewing a 27″ computer monitor (Acer Model #G276HL), participants were asked to move a computer cursor from left to right along a straight line to hit a displayed target at a distance of 22.4 cm (Fig. 7). Participants used a wireless mouse (Logitech Model #G703) on an extra wide mouse pad (31.5″ × 11.8″) to land on a stationary onscreen target. The sensitivity of the mouse was reduced to increase the physical movement distance in the Windows 10 operating system Mouse Settings menu under the ‘Pointer Options’ tab within the ‘Additional mouse settings’ menu. The pointer speed was selected as the third tick from the left under ‘Motion’ (third slowest setting) and ‘Enhance pointer position’ was deselected.

The movement was initiated with a participant mouse click that caused the cursor to disappear until the participant completed the movement and clicked again. At the completion of each movement, endpoint only visual feedback was provided on screen. View of the participant’s hand and arm movement was blocked by a rigid covering that did not contact the participant or impede movement of the computer mouse.

Participants wore noise canceling headphones (Mpow Model #BH366A) with Brownian noise playing to mask ambient auditory information. Participants were alerted with on-screen text when movement endpoints were off-screen past the target, backwards (i.e. initial movement direction was away from the target), or too short (i.e. ending the movement < 25% of the way to the target).

All participants completed three blocks of 100 successful trials, each under different noise conditions. Each movement block took about 6 min to complete. In one of the three blocks, movements were completed without added noise (baseline control). In the Low noise block, control noise with a standard deviation of 2.24 cm (10% of movement distance, Q = 5.04 cm^2^) was added, and noise with a standard deviation of 4.48 cm (20% of movement distance, Q = 20.11 cm^2^) was added in the High noise block. The noise was applied by computing a random number from a Gaussian distribution with the variance for that movement block and adding that shift to the landing position before the cursor was displayed to the participant. Each participant was randomly assigned one of six possible orders for the three noise conditions to appear.

To encourage participant engagement, a scoring system was implemented. Scores were displayed on-screen with points awarded for movements close to the target, and this was explained to participants. 200 points were awarded for direct target hits, 100 points for movement endpoints within 25 pixels (0.78 cm) of the target, and 50 points for within 100 pixels (3.12 cm) of the target. Participants were told that they could see how their score compared to the high score across all participants at the conclusion of the experiment.

### Analysis

To prepare the data for analysis, backwards and short movements were presumed to be accidental mouse clicks and removed from the movement records. Error during off-screen movements could not be measured and this resulted in a break in contiguous analyzable movement trials. The longest stretch of contiguous movement trials without an off-screen movement was extracted for each block of data to be analyzed. Steady-state trials in which initial parameter learning had stabilized were identified from the contiguous movement trials using a previously reported method ^10^. Across the 81 blocks of data collected, the average number of steady state trials analyzed was 93. One block resulted in only 11 steady state trials which was not enough to run the analyses. This participant was considered an outlier and their data across all three conditions were omitted. Empirical results presented include data from 26 participants. As in the simulated data, contiguous movement trials were analyzed using the conventional (Eq. 3), silver (Eq. 4), and analytic (Eq. 5) methods. For the silver method, the added noise on each trial was used, ignoring any baseline control noise generated by the participant. Likewise, for the analytic method the added control noise variance (Q) was used.

## Data Availability

Code used to simulate data, run the estimation analyses, and generate the figures, along with the simulated data used for estimation method analysis, and the empirical data are available here: 10.17605/OSF.IO/4VSMD.
